# Effect of Sodium Selenite and Hydroxy-Selenomethionine Supplementation in Hanwoo Cows on Reproductive Performance and Growth Performance of Their Offspring

**DOI:** 10.3390/ani16142258

**Published:** 2026-07-21

**Authors:** Young-Lae Kim, So-Hee Lee, Jeong-Keum Park, Gi-Hwal Son, Chang-Sik Choi, Chang-Woo Lee, Ju-Gyo Jeong, Jun Cui, Yun-He Zheng, Ji-Woo Choi, Young-Hwan We, Jong-Kook Bahn, Hyo-Won Seo, Jong-Suh Shin, Byung Ki Park, Min Ji Kim

**Affiliations:** 1 Department of Animal Science, Kangwon National University, Chuncheon 24341, Republic of Korea; yl1342@korea.kr (Y.-L.K.); seesohev@naver.com (S.-H.L.); choijhon2024@gmail.com (J.C.); yunhe010529@gmail.com (Y.-H.Z.); chlwldn0218@naver.com (J.-W.C.); weyounghan@hanmail.net (Y.-H.W.); niceab@naver.com (J.-K.B.); buffalopower@naver.com (H.-W.S.); jsshin@kangwon.ac.kr (J.-S.S.); animalpark@kangwon.ac.kr (B.K.P.); 2 Dairy Science Division, National Institute of Animal Science, Cheonan 31000, Republic of Korea; 3 Adisseo Asia Pacific Pte Ltd., Raffles Quay, Singapore 048581, Singapore; jenny.park@adisseo.com; 4 Nonghyup Livestock Research Center, Anseong 17558, Republic of Korea; oscar@naver.com; 5 Livestock Research Institute, Hoengseong 25266, Republic of Korea; ccs1512@korea.kr (C.-S.C.); k97112603@korea.kr (C.-W.L.); jjk7603@korea.kr (J.-G.J.)

**Keywords:** selenium, Hanwoo cow, Hanwoo calf, reproductive performance, growth performance

## Abstract

Selenium is an essential trace mineral that is involved in several biological processes, including antioxidant defense, immune function, and reproductive efficiency. Therefore, this study aimed to investigate the effects of selenium supplementation in Hanwoo cows on maternal reproductive performance and physiological responses, as well as the early growth and metabolic profiles of the offspring. Hanwoo cows (*n* = 30) were assigned to control (no supplement), sodium selenite (SS; 0.3 ppm/head/day), and hydroxy-selenomethionine (OH-SeMet; 0.3 ppm/head/day) treatment groups. Maternal reproductive performance and calf growth were examined. Cows in the OH-SeMet group had a significantly shorter postpartum interval and 4.25-fold higher conception rates than those in the control and SS groups. Plasma selenium concentrations were significantly higher in the OH-SeMet group at 3 months postpartum, with improved antioxidant status. Calves from OH-SeMet-supplemented cows had greater birth weights and higher plasma selenium, total immunoglobulin, and ghrelin concentrations. In addition, ruminal microbiota analysis revealed an increased Firmicutes-to-Bacteroidetes ratio in cows supplemented with OH-SeMet. In conclusion, dietary organic selenium supplementation in Hanwoo cows improved maternal reproductive efficiency and antioxidant status and promoted early growth in their offspring.

## Simple Summary

Mineral supplementation is an important practice in livestock production to improve the overall performance of animals. However, research on the potential carryover effects of maternal mineral supplementation on calf growth remains unknown. In this study, we showed that dietary supplementation with organic selenium, such as hydroxy-selenomethionine, improves maternal reproductive performance and has positive carryover effects on the performance of the offspring. Mechanistically, the improved reproductive performance of cows could be attributed to the higher bioavailability of organic selenium and the antioxidant effects of selenium. In addition, the improved performance of the offspring could be attributed to the transfer of selenium via the placenta during the fetal stage and colostrum after birth. Overall, our study highlights the importance of selenium supplementation on the performance of cows and their offspring.

## 1. Introduction

In pregnant cows, the peripartum period is critical for managing reproductive efficiency and ensuring calf health [1,2]. Appropriate nutritional management and supplementation during this period are essential for enhancing conception rates and supporting early calf growth and immune development, ultimately improving beef productivity and profitability. Among various peripartum management strategies, nutritional enhancement, including supplementation with vitamins, minerals, and trace elements, has gained considerable attention owing to its potential to alleviate metabolic stress, enhance immune competence, and improve reproductive performance in cows [2,3].

Selenium is an essential trace mineral for animals and plays a vital role in several physiological processes [4]. Notably, this trace mineral is involved in antioxidant defense, enhancement of immune function, and improvement of reproductive efficiency [5,6]. Selenium deficiency is associated with various health issues, including white muscle disease, mulberry heart disease, nutritional hepatic necrosis, and peripartum disorders (dystocia, retained placenta, postpartum paralysis, and early embryonic loss) [7]. In ruminants, selenium functions as a component of selenoenzymes, such as glutathione peroxidase (GPx), contributing to the neutralization of reactive oxygen species (ROS), enhanced immunity, and improved reproductive efficiency [7,8].

Research has shown that the estimated dietary requirements for selenium are approximately 300 µg/kg dry matter (DM) for beef cattle, 100 µg/kg DM for dairy cows, and 100 µg/kg DM for calves [9,10,11]. However, it is often difficult to meet these requirements using basal diets alone [12]. Selenium exists in both organic (selenium-containing amino acids, methylated compounds, selenoproteins, selenocysteine, and selenomethionine) and inorganic (metal selenides, elemental selenium, and selenates) forms, which affect its ruminal degradation and absorption efficiency [8,13]. Although inorganic selenium is absorbed primarily through passive diffusion and active transport in the small intestine, it exhibits low bioavailability, with limited storage as selenomethionine, and is excreted primarily via urine [14]. In contrast, organic selenium demonstrates high bioavailability owing to its active absorption and is stored in the body in the form of selenomethionine, which can later be utilized for selenoprotein synthesis and antioxidant activity, serving as a sustained source of selenium in the body [12,15].

While the beneficial effects of OH-SeMet on antioxidant status and immunity have been documented in various livestock species, research specifically targeting the reproductive longevity of Hanwoo cows and the trans-generational metabolic programming of their offspring remains limited. Given the unique metabolic characteristics of Hanwoo, it is crucial to establish breed-specific evidence. Therefore, this study is novel in that it comprehensively evaluates the link between maternal OH-SeMet supplementation and the calf’s growth performance during the critical pre-weaning period, providing a scientific basis for optimizing Hanwoo production systems.

Consequently, this study aimed to investigate the effects of selenium supplementation on the reproductive efficiency of Hanwoo cows and examine its carryover effects on the early growth of their calves. Specifically, we examined reproductive parameters and circulating reproductive hormone (estrogen and progesterone) concentrations in cows, as well as growth performance, total immunoglobulin (TIG) concentrations, and metabolic hormone (leptin, ghrelin, and growth hormone) concentrations in calves.

## 2. Materials and Methods

The experiment was conducted between June and December 2023. All procedures were reviewed and approved by the Institutional Animal Care and Use Committee of Kangwon National University (Chuncheon, Republic of Korea; approval no. KW-230227-3) and performed in accordance with institutional and national guidelines for animal experimentation.

### 2.1. Animals, Treatments, and Management

#### 2.1.1. Animals and Treatments

A total of 30 pregnant Hanwoo cows (body weight: 419.9 ± 64.4 kg; parity: 2.64 ± 1.45; month of age: 45.6 ± 20.8) were assigned to three dietary groups: control (no supplement), sodium selenite (SS; 0.3 ppm/head/day), and hydroxy-selenomethionine (OH-SeMet; 0.3 ppm/head/day). Selenium dosage was set at 0.3 mg/kg [16] based on the maximum allowable limit of selenium (0.5 mg/kg) in compound feeds, as regulated by the Ministry of Agriculture, Food and Rural Affairs (MAFRA) in South Korea. Notably, this dosage ensured that the total selenium concentration, including the background levels of the basal ingredients, remained within the legal safety thresholds. The cows were supplemented with selenium from 3 months before calving until 3 months postpartum. To evaluate offspring responses, 30 calves (birth weight: 28.16 ± 4.16 kg) born to these cows were also included in the study. Selenium supplementation was provided only to the cows; calves did not receive direct supplementation, and their outcomes were evaluated as a function of maternal selenium transfer.

#### 2.1.2. Management

Pregnant Hanwoo cows were fed concentrates and rice straw, whereas Hanwoo calves were fed calf starters and timothy.

All experimental animals were housed in group pens according to their respective treatment groups. Cows were fed concentrates and rice straw at 4 kg/day and 7 kg/day, respectively. Feed was offered twice daily at 07:00 and 16:00, while water, mineral blocks, and sodium bicarbonate were provided ad libitum. To facilitate adaptation to solid feed, calves were provided with a concentrated starter and timothy hay starting from 3 weeks of age. Feeding levels were determined according to the Korean Feeding Standard for Hanwoo Cattle [17]. The chemical compositions of the experimental diets are shown in Table 1. Selenium premixes were formulated to contain equivalent levels of other minerals (Cu, Fe, Mn, Co, Zn, and I), vitamins (A, D_3_, and E), and *Clostridium butyricum*, with selenium concentration being the only variable among treatments. Cornmeal was used as a carrier to ensure a balanced nutrient supply across the treatments.

### 2.2. Measurements

#### 2.2.1. Chemical Composition of Experimental Diets

The chemical composition of the experimental diets was analyzed according to the methods of AOAC [18]. Neutral detergent fiber and acid detergent fiber were determined according to the method of Van Soest et al. [19] using a fiber analyzer (Ankom 2000, Ankom Technology, Macedon, NY, USA).

#### 2.2.2. Reproductive Efficiency

The interval to estrus return was measured using a neck-mounted estrus detection device (HANUSEN, Hankook IoT Corp., Gimcheon, Republic of Korea) attached to the left side of the neck, which automatically recorded the activity and behavioral changes associated with estrus. Data were transmitted to the herd management system for validation and analysis. In addition, the conception rate and number of inseminations per conception were recorded for each cow.

#### 2.2.3. Growth Performance

The birth weight and body weight of the calves at 3 months of age were measured using a digital scale (Newton NT-501A, CAS Korea, Seongnam, Republic of Korea) and used to calculate the average daily gain (ADG) of the calves. Feed intake was calculated based on the amount of feed offered and the remaining feed the next morning. Feed conversion ratio (FCR) was calculated as the ratio of dry matter intake (DMI) to average daily gain (ADG) for each experimental period.

#### 2.2.4. Blood Collection and Plasma Metabolite Profiling

For the analysis of plasma metabolites, blood samples were collected from cows three times: 3 months before calving, immediately after calving, and 3 months postpartum. Blood samples were collected from calves at 3 months of age for further analysis. Blood was drawn from the jugular vein using 10 mL vacutainers (Becton Dickinson Co., Franklin Lakes, NJ, USA). For plasma analysis, samples were collected in tubes containing heparin, left to stand at 4 °C for 6 h, and centrifuged at 1250× *g* for 15 min to separate the plasma. Plasma glucose, blood urea nitrogen (BUN), albumin, non-esterified fatty acid (NEFA), triglyceride, cholesterol, creatinine, aspartate aminotransferase (AST), alanine aminotransferase (ALT), γ-glutamyl transferase (GGT), calcium (Ca), inorganic phosphate (IP), and magnesium (Mg) concentrations were determined using an automatic biochemical analyzer (Hitachi 7020, Hitachi Ltd., Tokyo, Japan).

#### 2.2.5. Plasma Selenium, GPx, and Hormone Analysis

Plasma Selenium and GPx concentrations in cows and calves were analyzed using ELISA kits (selenium: EK760208, GPx: EK760015; AFG Bioscience, Northbrook, IL, USA) according to the manufacturer’s protocol.

ELISA kits were used to analyze the concentrations of progesterone and estrogen in the plasma of the cows (Estrogen: MBS2610579, Progesterone: MBS766057; MYBioSource, San Diego, CA, USA). In addition, TIG and growth-related hormone (leptin, ghrelin, and growth hormone) concentrations in the plasma of calves were analyzed using ELISA kits (TIG: MBS738656, Leptin: MBS703026, Ghrelin: MBS2024801, Growth: MBS2086954; MYBioSource, San Diego, CA, USA).

#### 2.2.6. DNA Extraction and 16S rRNA Gene Sequencing

Rumen fluid was collected using a stomach tube before morning feeding, transferred to 50 mL tubes, and stored at −80 °C until further analysis.

Genomic DNA was extracted for 16S rRNA gene amplification using the QIAamp^®^ Fast DNA Stool Mini Kit (Cat. No. 51604; QIAGEN, Hilden, Germany), according to the manufacturer’s instructions. Next-generation sequencing (NGS) was performed by HuN Biome Inc. (Seoul, Republic of Korea).

Sequencing libraries were prepared according to the Illumina 16S Metagenomic Sequencing Library protocol to amplify the V3 and V4 regions. Primary PCR amplification was performed by mixing 2 ng of template gDNA with 5× reaction buffer, 1 mM dNTP mix, 500 nM of each universal F/R PCR primer, and Herculase II fusion DNA polymerase (Catalog #600675; Agilent Technologies, Santa Clara, CA, USA). The PCR conditions were as follows: initial denaturation at 95 °C for 3 min, 25 cycles of denaturation at 95 °C for 30 s, annealing at 55 °C for 30 s, extension at 72 °C for 30 s, and final extension at 72 °C for 5 min. The universal primer pairs containing the Illumina adapter overhang sequences used for the first amplification were as follows:

V3-F: 5′-TCGTCGGCAGCGTCAGATGTGTATAAGAGACAGCCTACGGGNGGCWGCAG-3′

V4-R: 5′-GTCTCGTGGGCTCGGAGATGTGTATAAGAGACAGGACTACHVGGGTATCTAATCC-3′

The primary PCR products were purified using AMPure beads (Agencourt Bioscience, Beverly, MA, USA). After purification, 2 µL of the first PCR product was subjected to a second PCR amplification using Nextera XT Indexed Primers to construct the final library. The cycling conditions for the second PCR were identical to those of the first but were limited to 10 cycles. The resulting PCR products were purified using AMPure beads.

The final purified products were quantified using qPCR according to the qPCR Quantification Protocol Guide (KAPA Library Quantification kits for Illumina Sequencing platforms, Roche, MA, USA), and their quality was assessed using TapeStation D1000 ScreenTape (Agilent Technologies, Waldbronn, Germany). Paired-end (2 × 300 bp) sequencing was performed using a MiSeq™ platform (Illumina, San Diego, CA, USA) at Macrogen (Seoul, Republic of Korea).

### 2.3. Statistical Analysis

All statistical analyses were performed using IBM SPSS Statistics (Statistical Package for the Social Sciences, version 29; SPSS Inc., Chicago, IL, USA). Data are presented as mean ± standard error. One-way analysis of variance (ANOVA) was used to compare the effects of the treatments on the reproductive performance of cows and the growth performance of calves at a single time point. Plasma parameters were analyzed using two-way repeated-measures ANOVA with treatment (control, SS, and OH-SeMet) as the between-subject factor and time point (3 months before calving, immediately after calving, and 3 months postpartum) as the within-subject factor. The interaction between treatment and time points (A × B) was also evaluated. The normality of the data and homogeneity of variance were verified using the Shapiro–Wilk test and Levene’s test, respectively. Post hoc comparisons were conducted using Tukey’s test. Statistical significance was set at *p* < 0.05. Binary logistic regression analysis was used to evaluate the effects of treatment on the conception rate.

## 3. Results

### 3.1. Hanwoo Cows

#### 3.1.1. Reproductive Efficiency

In this study, we investigated the effects of selenium supplementation on the reproductive efficiency of Hanwoo cows (Table 2 and Table 3).

Although not statistically significant (*p* = 0.122), the interval from calving to the first postpartum estrus was shorter in the OH-SeMet group than in the control and SS groups. In addition, the number of services per conception was lower in the OH-SeMet group than in the control and SS groups, although the difference was not statistically significant. Binary logistic regression analysis indicated that cows in the OH-SeMet group were approximately 4.25 times more likely to conceive than those in the control and SS groups (*p* < 0.05).

#### 3.1.2. Plasma Selenium, GPx, and Hormone Concentration

**Figure 1:**
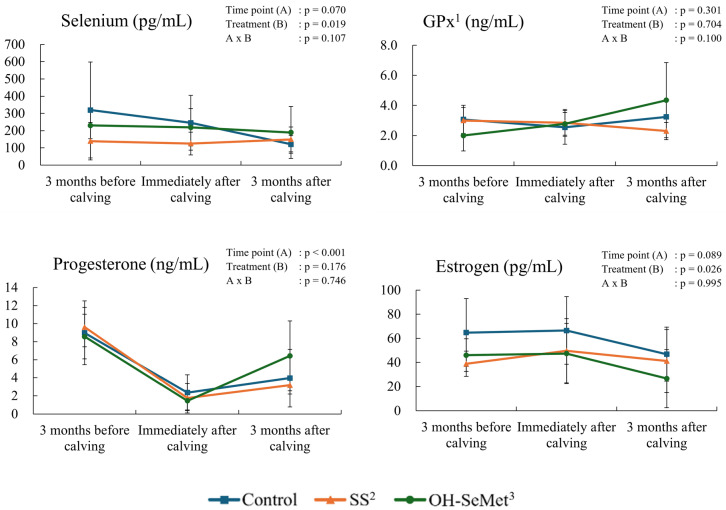
Effect of sodium selenite and hydroxy-selenomethionine supplementation on plasma selenium and glutathione peroxidase concentrations in Hanwoo cows at 3 months before and after calving. ^1^ GPx: glutathione peroxidase; ^2^ SS: sodium selenite; ^3^ OH-SeMet: hydroxy-selenomethionine. Data are presented as mean ± standard error (*n* = 10 per treatment). Data were analyzed using two-way repeated-measures ANOVA with Tukey’s post hoc test.

Figure 1 shows the effects of selenium supplementation on plasma selenium, GPx, and hormone (progesterone and estrogen) concentration in Hanwoo cows.

Plasma selenium and GPx concentrations were lower in the OH-SeMet group than in the control group at 3 months prepartum. However, both selenium and GPx concentrations were higher in the OH-SeMet group than in the control and SS groups at 3 months postpartum. Among the treatment groups, selenium concentration was highest in the OH-SeMet group. Although GPx activity was not significantly affected by time point or treatment, it was higher in the OH-SeMet group than in the other groups at 3 months postpartum (*p* = 0.100). Although no significant differences were observed in plasma progesterone concentrations among the groups at 3 months prepartum, progesterone concentrations decreased rapidly immediately after parturition. At 3 months postpartum, progesterone concentrations were higher in the OH-SeMet group than in the control and SS groups. Plasma estrogen concentrations were significantly lower in the OH-SeMet group than in the control group immediately after parturition and at 3 months postpartum (*p* < 0.05).

#### 3.1.3. Plasma Metabolites

**Figure 2:**
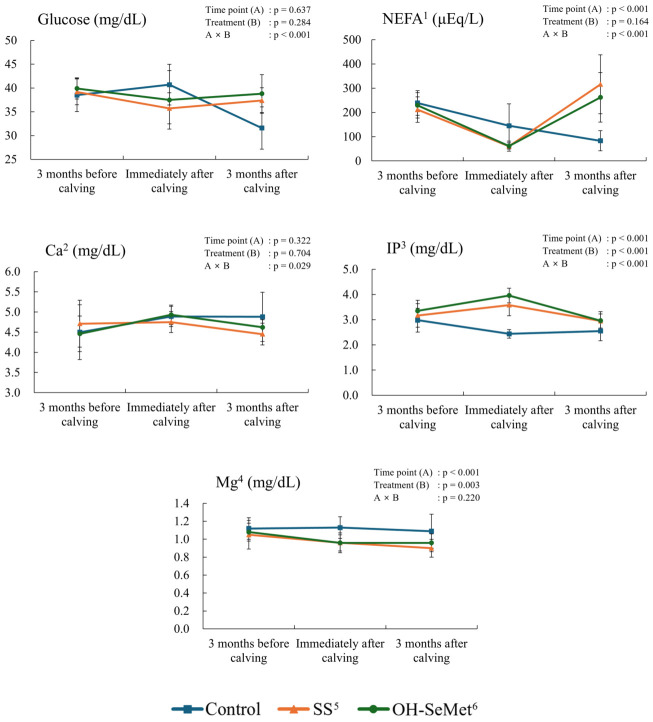
Effect of sodium selenite and hydroxy-selenomethionine supplementation on plasma metabolite concentrations in Hanwoo cows. ^1^ NEFA: non-esterified fatty acid; ^2^ Ca: calcium; ^3^ IP: inorganic phosphate; ^4^ Mg: magnesium; ^5^ SS: sodium selenite; ^6^ OH-SeMet: hydroxy-selenomethionine. Data are presented as mean ± standard error (*n* = 10 per treatment). Data were analyzed using two-way repeated-measures ANOVA with Tukey’s post hoc test.

In this study, we investigated the effects of selenium supplementation on plasma metabolites in Hanwoo cows (Figure 2 and Figure S1).

Time point and treatment exhibited a significant interactive effect on plasma glucose concentrations (*p* < 0.001), with the SS and OH-SeMet groups showing lower than the control group at 3 months postpartum. In addition, NEFA concentration was significantly influenced by time point (*p* < 0.001) and its interaction with treatment (*p* < 0.001). Notably, time point and treatment exhibited a significant interactive effect on plasma Ca concentrations (*p* < 0.05), with lower in the SS and OH-SeMet groups than in the control group at 3 months postpartum. Plasma IP concentrations were highest in the OH-SeMet group immediately after calving and remained consistently higher than those in the control group at 3 months postpartum. In contrast, plasma Mg concentrations were lower in the SS and OH-SeMet groups than in the control group at all time points (*p* < 0.01).

#### 3.1.4. Ruminal Microbiota (Phylum)

**Figure 3:**
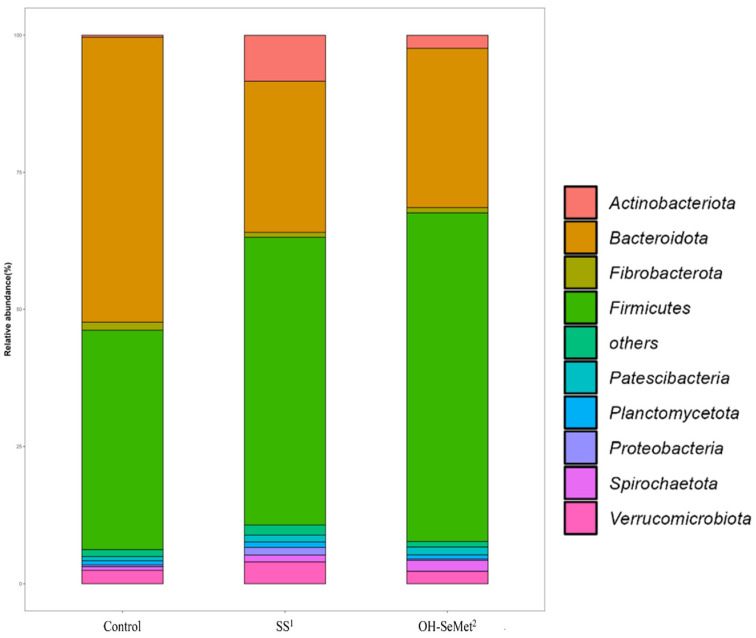
Effect of sodium selenite and hydroxy-selenomethionine supplementation on rumen microbiota level in Hanwoo cows. ^1^ SS: sodium selenite; ^2^ OH-SeMet: hydroxy-selenomethionine.

In this study, we investigated the effects of selenium supplementation on the relative abundance of ruminal microbiota at the phylum level in Hanwoo cows (Table 4, Figure 3).

Notably, the relative abundance of *Firmicutes* was significantly higher in the SS and OH-SeMet groups than in the control group (*p* < 0.01). Conversely, the proportion of *Bacteroidota* was significantly lower in the SS and OH-SeMet groups than in the control group (*p* < 0.01). In addition, the proportion of *Patescibacteria* was significantly higher in the SS and OH-SeMet groups than in the control group (*p* < 0.05).

### 3.2. Hanwoo Calves

#### 3.2.1. Growth Performance

Table 5 shows the effects of selenium supplementation on the growth characteristics of suckling Hanwoo calves.

Notably, the birth weights of calves were significantly higher in the OH-SeMet group than in the control group (*p* < 0.05). Although not statistically significant, body weights at 3 months of age were higher in the SS and OH-SeMet groups than in the control group (*p* = 0.106). ADG was highest in the OH-SeMet group, followed by the SS and control groups.

#### 3.2.2. Plasma Selenium, GPx, TIG, and Hormone Concentrations

Table 6 shows the effects of selenium supplementation in Hanwoo cows on the plasma concentrations of selenium, GPx, TIG, and hormones in the suckling calves.

Plasma selenium and TIG concentrations were higher in the OH-SeMet group than in the control and SS groups. Although not statistically significant, plasma GPx concentrations were higher in the SS and OH-SeMet groups than in the control group. In addition, plasma leptin concentrations were lower in the OH-SeMet group than in the control and SS groups. In contrast, ghrelin concentrations were significantly higher in the OH-SeMet group than in the control and SS groups (*p* < 0.001). However, no significant intergroup differences were observed in growth hormone concentrations.

#### 3.2.3. Plasma Metabolite

Table 7 presents the effects of selenium supplementation in Hanwoo calves on the plasma metabolome of suckling calves.

Plasma NEFA concentration was lower in the OH-SeMet group than in the control and SS groups. In addition, plasma cholesterol concentrations were significantly lower in the OH-SeMet group than in the control group (*p* < 0.05). GGT concentrations tended to be higher in the OH-SeMet group than in the control and SS groups, whereas AST concentrations were lower in both the SS and OH-SeMet groups than in the control group.

## 4. Discussion

### 4.1. Hanwoo Cows

#### 4.1.1. Reproductive Efficiency

The higher reproductive efficiency observed in the OH-SeMet group suggests that OH-SeMet supplementation positively impacts the reproductive performance of Hanwoo cows. In particular, cows in the OH-SeMet group exhibited a shorter postpartum estrus interval and higher conception rates than those in the control and SS groups. Selenium may protect ovarian function through antioxidant defense mechanisms and enhance estrus recovery and conception success by promoting luteal cell survival and increasing progesterone secretion [11,20]. Notably, OH-SeMet has higher bioavailability and greater tissue retention than SS, which may contribute to more effective endocrine regulation and reproductive tissue recovery [8,21]. Consistent with these findings, Khalili et al. [22] reported that supplementation with 0.5 ppm organic selenium improved days to postpartum estrus and number of services per conception in dairy cows. Overall, these results suggest that OH-SeMet supplementation can positively influence reproductive efficiency, possibly due to the high bioavailability of organic selenium. Moreover, the higher first conception rate observed in the organic Se group may contribute to reduced production costs, such as feed expenses and artificial insemination fees, before the second service.

#### 4.1.2. Plasma Selenium, GPx, and Hormone Concentration

Plasma selenium concentrations were consistently higher in the OH-SeMet group than in the other groups during the postpartum period, which may indicate effective retention and utilization of selenium in the body tissues. GPx, a representative selenoenzyme, plays a key role in antioxidant defense by removing ROS, thereby protecting cell membranes and DNA from oxidative damage [13,23]. Although no clear treatment effect was evident on GPx activity, the observed tendency over time suggests that organic selenium may support a sustained antioxidant response during the peripartum period, potentially through improved selenium utilization and retention [6,24]. Collectively, these findings suggest that OH-SeMet supplementation is effective in maintaining antioxidant defense systems and improving the nutritional status of selenium in cows.

In addition, the rapid decline in progesterone concentrations immediately after calving is considered a typical physiological response, as progesterone secretion from the corpus luteum and placenta rapidly declines once pregnancy ends, allowing the initiation of uterine involution and the transition to lactation-related endocrine regulation [25,26]. Moreover, the elevated progesterone concentration in the OH-SeMet group at 3 months postpartum suggests a rapid recovery of the luteal phase, possibly indicating the resumption of ovulation and the normalization of the estrous cycle [26]. This may have contributed to the shorter days to estrus return observed in the OH-SeMet group compared with the control and SS groups (Table 2).

Estrogen, a key steroid hormone, plays essential roles in late gestation, fetal maturation, initiation of parturition, regulation of uterine contractions, and postpartum return to estrus, with its levels typically increasing immediately before calving [27]. In the present study, although the OH-SeMet group exhibited lower plasma estrogen concentrations, cows in this group had the shortest interval to return to estrus and the highest conception rate (Table 2). The apparent discrepancy between estrogen concentrations and estrus resumption suggests that factors beyond circulating estradiol concentrations, such as LH pulse recovery and ovarian responsiveness, may have played a role, which is consistent with previous reports on early lactation cows [28,29]. Similarly, Cerny et al. [30] reported that selenium supplementation, including organic forms, tended to increase progesterone concentrations in both serum and follicular fluid but had no effect on estradiol concentrations. Selenum protects ovarian and luteal cells from oxidative damage through selenoproteins, such as GPx, which maintain the cellular redox balance and support steroidogenic enzyme activity [31,32]. Therefore, the improved reproductive outcomes in the OH-SeMet group may be attributed to enhanced redox homeostasis and steroidogenic function in ovarian tissues.

Therefore, OH-SeMet supplementation supported normal physiological transitions in reproductive hormones and contributed to the restoration of hormonal balance with normal physiological changes in reproductive hormones and may positively contribute to the restoration of hormonal balance, particularly during the recovery of the luteal phase after parturition. These outcomes reflect the beneficial effects of selenium on reducing oxidative stress and improving overall metabolic recovery, thereby indirectly contributing to improved reproductive function.

#### 4.1.3. Plasma Metabolites

In this study, the increase in plasma glucose concentration in the control group immediately after calving was considered a response to parturition stress. Conversely, the decrease in plasma glucose concentrations in the SS and OH-SeMet groups suggests that supplementation with these compounds may mitigate calving stress. Although SS can contribute to alleviating oxidative stress, its bioavailability is relatively low because it is reduced and precipitated within the rumen, thereby limiting its storage capacity in tissues [33]. In contrast, OH-SeMet can be stored in tissues over the long term because of its structural similarity to methionine. OH-SeMet exerts continuous antioxidant and metabolic buffering effects, even during periods of negative energy balance, such as the immediate post-calving period [34]. Therefore, the gradual decrease and rapid normalization of glucose concentrations in the OH-SeMet group suggest that OH-SeMet contributes to hepatic metabolic stability and the regulation of energy mobilization, thereby maintaining metabolic homeostasis.

The control group exhibited higher plasma NEFA concentrations and peak glucose concentrations immediately after calving, reflecting a typical acute stress response and rapid fat mobilization triggered by parturition. In contrast, cows in the SS and OH-SeMet groups exhibited significantly lower NEFA concentrations, suggesting that selenium supplementation effectively mitigated the initial metabolic shock during parturition. Plasma glucose and NEFA concentrations decreased in the control group at 3 months after calving, indicating a potential decline in metabolic activity. In contrast, cows in the SS and OH-SeMet groups exhibited a marked increase in NEFA concentrations and higher and more stable plasma glucose concentrations than those in the control group. Importantly, the elevated NEFA concentrations in the SS and OH-SeMet groups represented an active and healthy mobilization of energy reserves rather than pathological lipidosis. Selenium-induced upregulation of mitochondrial β-oxidation likely facilitates the efficient conversion of mobilized fatty acids into usable energy, thereby maintaining superior glucose homeostasis [6,35]. Overall, these results suggest that selenium supplementation, particularly OH-SeMet, optimizes the metabolic transition in Hanwoo cows by preventing excessive stress-induced mobilization during calving and supporting robust energy turnover during the peak physiological demand period, which is 3 months after calving.

Although previous studies have investigated the relationships between macrominerals (Ca, P, and Mg) and trace elements, such as selenium, in pregnant heifers [36,37], direct evidence of the effects of selenium supplementation on mineral metabolism remains limited. In the present study, plasma Ca, P, and Mg concentrations were influenced by selenium supplementation, although the underlying mechanisms remain unclear. Plasma Ca and Mg concentrations were slightly lower in the selenium-supplemented groups, whereas IP concentrations were higher in the OH-SeMet group and remained elevated postpartum. Collectively, these findings suggest that selenium supplementation may modulate mineral metabolism; however, these effects should be regarded as speculative and warranting further investigation.

In this study, OH-SeMet supplementation in Hanwoo cows was instrumental in optimizing periparturient metabolic transition, effectively facilitating robust energy turnover. Temporal glucose kinetics, characterized by a gradual decline followed by expedited normalization, underscore the role of OH-SeMet in enhancing hepatic metabolic stability and modulating energy mobilization to maintain systemic homeostasis. Although evidence suggests that this selenium source may exert a modulatory effect on mineral metabolism, these findings remain preliminary and require further investigation.

#### 4.1.4. Ruminal Microbiota (Phylum)

As illustrated in Figure 3, these shifts in the microbial community composition indicated that selenium supplementation significantly modulated the ruminal ecosystem. Specifically, the observed increase in the *Firmicutes*/*Bacteroidota* (F/B) ratio (Figure 3) is closely associated with enhanced energy harvesting capacity in ruminants [38]. *Firmicutes* are known to be more involved in fiber degradation and the production of butyrate, which serves as a major energy source for the host. Therefore, the higher abundance of *Firmicutes* in the selenium-treated groups may suggest a more efficient breakdown of structural carbohydrates, potentially providing additional energy to meet the high metabolic demands of the cows during the periparturient period. A decrease in *Bacteroidota* abundance represents a structural reorganization of the microbial community, potentially partitioning more nutrients toward energy-efficient pathways [39,40]. These changes are considered to be the result of improved antioxidant status within the rumen due to selenium supplementation, which alleviates oxidative stress and creates a more favorable environment for microbes. In particular, this increase in ruminal energy-harvesting potential serves as a critical physiological foundation supporting the rapid recovery of plasma glucose and active mobilization of NEFA observed at 3 months postpartum (Figure 2). The elevated NEFA concentrations in the treatment groups during this period were interpreted as a healthy metabolic turnover supported by an improved ruminal energy supply rather than a signal of pathological energy deficiency [6,35]. In the present study, the observed changes in ruminal microbial composition and systemic metabolic indicators may reflect a more favorable metabolic adaptation during the peripartum period. Collectively, selenium supplementation was associated with the metabolic and reproductive improvements observed in Hanwoo cows, potentially through improved energy partitioning supporting maternal recovery and lactation.

### 4.2. Hanwoo Calves

#### 4.2.1. Growth Performance

These results suggest that supplementation with organic selenium positively affects the birth weight and growth performance of suckling calves. In particular, the higher birth weight observed in the OH-SeMet group was likely related to the maternal transfer of selenium. OH-SeMet has high bioavailability and a superior placental transfer rate, which may contribute to enhanced fetal growth and development. In addition, the higher body weight of calves in the OH-SeMet group at 3 months of age may also be attributed to the transfer of selenium through milk, which positively affected calf growth.

Moreover, the high ADG observed in the OH-SeMet group further suggests that organic selenium may alleviate oxidative stress and improve metabolic efficiency, thereby promoting protein synthesis and growth in calves.

Furthermore, the higher bioavailability of organic selenium may be explained by the fact that SeMet shares the same amino acid transport system as methionine, allowing efficient transfer to the fetus via the placenta [41,42]. The superior placental transfer rate of organic selenium is a pivotal factor in promoting fetal tissue formation and growth during late gestation [34]. Although not statistically significant (*p* = 0.106), calves in the OH-SeMet group had higher body weights at 3 months and ADG than those in the other groups [13], which may be closely linked to the metabolic regulatory functions of selenium [43]. As an essential component of iodothyronine deiodinases, selenium facilitates the conversion of thyroxine (T4) to active triiodothyronine (T3), which promotes robust growth in calves by regulating the basal metabolic rate, mitochondrial β-oxidation, and protein synthesis [44]. Notably, the dry matter intake (DMI) of calves in the OH-SeMet group was 0.94 kg/day, representing an approximately 16% increase compared with that in the control group (0.81 kg/day). This increase in feed intake in the OH-SeMet group may be attributed to the endocrine-mediated effect of selenium supplementation. However, further research involving direct thyroid hormone analysis is necessary to investigate the endocrine effects of selenium. Previous studies have indicated that maternal supplementation with organic selenium during late gestation and early lactation increases the visceral mass of offspring and assists in the proliferation of crypt cells within the gastrointestinal tract [45,46]. This gastrointestinal development enhances nutrient absorption capacity, providing a physiological foundation for calves to consume more feed and nutrients for growth prior to weaning.

Therefore, supplementation of maternal diet with OH-SeMet significantly improved birth weight through efficient placental selenium transfer and contributed to optimizing the early growth performance and metabolic efficiency of Hanwoo calves by inducing thyroid hormone activation and gastrointestinal development.

#### 4.2.2. Plasma Selenium, GPx, TIG, and Hormone Concentrations

Plasma selenium concentration was markedly higher in the OH-SeMet group, which may be related to the stable absorption of OH-SeMet in the digestive tract and its nonspecific incorporation into proteins via the methionine metabolic pathway. Similarly, Hendawy et al. [14] and Sun et al. [47] reported that organic selenium has a higher retention rate in the body than inorganic selenium. However, the lack of statistical significance in certain parameters may be due to the substantial individual variation in blood selenium and antioxidant concentrations observed among the animals. Previous research has shown that metabolic responses to selenium supplementation tend to be highly variable in ruminants and are often influenced by individual factors, previous selenium exposure, and basal dietary concentrations [13], indicating that selenium transfer and subsequent metabolic utilization are subject to significant individual-specific differences. Although not statistically significant, GPx concentrations were higher in the SS and OH-SeMet groups than in the control group. This is likely because selenium acts as a key structural component of GPx, enhancing antioxidant enzyme activity [8].

Leptin is primarily secreted from adipose tissue and is strongly associated with body fat accumulation, serving as a physiological indicator of energy balance and growth status in calves [48]. The reduced leptin concentrations observed in the OH-SeMet group may reflect decreased adipose deposition during the early growth stage, suggesting that dietary OH-SeMet supplementation promotes nutrient utilization for lean tissue accretion rather than fat storage. Given that excessive oxidative stress can inhibit the functional integrity of hypothalamic neurons responsible for energy homeostasis, selenium-mediated mitigation of oxidative damage may normalize or activate the neuroendocrine signaling mechanisms of both leptin and ghrelin [49]. Recent studies have demonstrated that lowering oxidative stress within specialized cells can directly stimulate the secretion of metabolic hormones, such as ghrelin, thereby enhancing appetite regulation and energy partitioning efficiency [50]. Therefore, lower leptin concentrations in OH-SeMet-supplemented calves should not be regarded as a negative outcome but as a potential indicator of improved growth efficiency, optimized neuroendocrine signaling, and reduced fat accumulation.

Elevated plasma ghrelin concentrations in the OH-SeMet group suggest that selenium supplementation modulates key orexigenic hormones associated with growth. This increase may be attributed to reduced oxidative stress and enhanced endocrine homeostasis, which provide a favorable physiological environment for ghrelin secretion [8]. Given that ghrelin stimulates appetite, gastric emptying, and growth hormone secretion [51,52], these results are consistent with the higher calf starter intake observed in the OH-SeMet group (Table 4). Therefore, maternal OH-SeMet supplementation appears to optimize offspring growth performance by enhancing appetite regulation and nutrient utilization efficiency in offspring.

Our findings suggest that maternal supplementation with OH-SeMet during gestation and its subsequent transfer via the fetus and colostrum are key mechanisms for early immune establishment and growth promotion in calves. However, a limitation of this study is that the maternal transfer effects were evaluated only at 3 months of age. Although this period is appropriate for identifying early growth indicators, the high individual variation observed in certain parameters presents challenges in achieving statistical significance. Therefore, future research should include long-term follow-up studies of the growth and fattening periods after 3 months of age to verify the metabolic effects of early selenium transfer on the final carcass performance and meat quality of Hanwoo cattle.

#### 4.2.3. Plasma Metabolite

Selenium supplementation facilitates fatty acid oxidation in the liver, effectively redirecting circulating NEFA to the hepatic tissues for energy production [53,54]. This enhanced hepatic uptake and oxidation, driven by optimized metabolic coordination, resulted in decreased blood NEFA concentrations. Overall, these findings suggest that OH-SeMet promotes nutrient partitioning toward lean tissue accretion by improving the coordination between lipid mobilization and energy metabolism. Furthermore, the decrease in cholesterol concentration in the SS and OH-SeMet groups indicates that selenium may indirectly regulate cholesterol biosynthesis by inhibiting the activity of HMG-CoA reductase (3-hydroxy-3-methylglutaryl-coenzyme A reductase) through antioxidant pathways. Importantly, this effect tends to be more pronounced with organic selenium and is closely related to increased selenium concentrations in liver tissue [55,56]. Collectively, these findings indicate that OH-SeMet may improve lipid homeostasis by modulating cholesterol synthesis and degradation pathways, thereby optimizing the physiological energy balance for growth in suckling calves. The elevated plasma GGT concentration in the OH-SeMet group may reflect increased detoxification activity and enhanced glutathione metabolism [57]. Although GGT is generally regarded as a marker of hepatic damage, the lower AST concentration observed in the OH-SeMet group suggests that the increase in GGT activity may reflect enhanced metabolic activation and detoxification capacity rather than hepatocellular injury. Overall, these results suggest that maternal OH-SeMet supplementation may exert carryover effects in suckling calves, particularly by modulating lipid metabolism and enhancing hepatic metabolic function and antioxidant capacity.

## 5. Conclusions

OH-SeMet is characterized by superior bioavailability and tissue retention compared with inorganic selenium, enabling more efficient utilization in metabolic and reproductive processes. In the present study, supplementation with 0.3 ppm/day OH-SeMet during the peripartum period in Hanwoo cows improved both maternal and offspring outcomes.

In Hanwoo cows, OH-SeMet supplementation significantly increased the conception rate, maintained higher plasma selenium concentrations, and reduced circulating estrogen concentrations, thereby supporting reproductive efficiency. In addition, OH-SeMet supplementation optimized the ruminal microbial community by increasing the F:B ratio, which is thought to have a positive impact on energy harvesting efficiency. These shifts in the microbial community provide a physiological basis for a healthy metabolic turnover in the OH-SeMet group. Unlike the stagnant metabolic state of the control group, the OH-SeMet group showed stable glucose homeostasis and active NEFA mobilization at 3 months postpartum, indicating a more resilient metabolic transition. Notably, OH-SeMet supplementation in maternal diet enhanced birth weight and markedly elevated plasma ghrelin concentrations in Hanwoo calves, suggesting improved appetite regulation and early growth.

In conclusion, these findings demonstrate that dietary supplementation with OH-SeMet (0.3 ppm/day) is an effective nutritional strategy for improving reproductive performance in Hanwoo cows and promoting growth and metabolic health in their calves.

## Figures and Tables

**Table 1:** Chemical composition of experimental diets (DM-basis).

Item (%)	Hanwoo Cow		Hanwoo Calf	
	Concentrate	Rice Straw	Concentrate	Timothy
Dry matter	90.01 ± 0.72	86.40 ± 0.95	91.40 ± 0.76	93.60 ± 0.81
Crude protein	15.79 ± 0.43	6.24 ± 0.31	19.23 ± 0.43	13.25 ± 0.32
Ether extract	3.72 ± 0.45	1.08 ± 0.46	3.12 ± 0.73	1.50 ± 0.12
Crude ash	7.15 ± 1.55	13.10 ± 0.48	8.59 ± 0.86	6.84 ± 0.11
Crude fiber	5.85 ± 1.29	38.72 ± 0.68	12.29 ± 4.11	43.06 ± 0.55
Neutral detergent fiber	36.44 ± 6.31	71.62 ± 0.71	34.54 ± 3.74	65.17 ± 0.93
Acid detergent fiber	20.25 ± 0.79	49.89 ± 0.66	17.89 ± 1.52	38.35 ± 0.43

**Table 2:** Effect of sodium selenite and hydroxy-selenomethionine supplementation on reproductive efficiency in Hanwoo cows.

Item		Control	SS ^1^	OH-SeMet ^2^	*p*-Value
Day to postpartum estrous (d)		37.38 ± 8.75	49.07 ± 23.10	34.11 ± 10.94	0.122
Number of service per conception		1.33 ± 0.58	1.63 ± 0.74	1.17 ± 0.41	0.404
Conception rate (%)	1st	22	36	63	-
	2nd	33	64	75	-

^1^ SS: sodium selenite; ^2^ OH-SeMet: hydroxy-selenomethionine. Data are presented as means ± standard error (*n* = 10 per treatment). Data were analyzed using one-way ANOVA with Tukey’s post hoc test.

**Table 3:** Results of binary logistic regression analysis for conception rate in Hanwoo cows.

Variable	Odds Ratio (Exp(B))	95% Confidence Interval	*p*-Value
Treatment group	4.249	1.022–17.662	0.047
Day to postpartum estrous (d)	1.024	0.970–1.082	0.390
Number of service per conception	0.384	0.113–1.303	0.125

Treatment group reference: control. Odds ratios estimated from the binary logistic regression predicting conception rates.

**Table 4:** Effects of sodium selenite and hydroxy-selenomethionine supplementation on ruminal microbiota (phylum) level in Hanwoo cows.

Item (%)	Control	SS ^1^	OH-SeMet ^2^	*p*-Value
*Firmicutes*	39.97 ± 4.37 ^b^	52.43 ± 1.19 ^a^	59.91 ± 3.90 ^a^	0.001
*Actinobacteriota*	0.37 ± 0.14	8.40 ± 11.36	2.37 ± 2.19	0.371
*Bacteroidota*	51.97 ± 3.89 ^a^	27.54 ± 7.31 ^b^	29.02 ± 2.92 ^b^	0.002
*Verrucomicrobiota*	2.46 ± 0.58	4.00 ± 1.58	2.27 ± 0.56	0.156
*Patescibacteria*	0.78 ± 0.18 ^b^	1.28 ± 0.18 ^a^	1.42 ± 0.22 ^a^	0.016
*Planctomycetota*	0.76 ± 0.24	1.00 ± 0.39	0.77 ± 0.40	0.653
*Fibrobacterota*	1.47 ± 0.94	0.88 ± 0.58	0.97 ± 0.63	0.602
*Spirochaetota*	0.67 ± 0.12	1.24 ± 0.32	1.99 ± 1.56	0.285
*Proteobacteria*	0.31 ± 0.08	1.39 ± 0.88	0.25 ± 0.06	0.059
Others	1.25 ± 0.29	1.83 ± 0.62	1.01 ± 0.24	0.122

^a.b^ Means with different superscripts in the same row are significantly different (*p* < 0.05). ^1^ SS: sodium selenite 0.3 ppm; ^2^ OH-SeMet: hydroxy-selenomethionine 0.3 ppm. Data are presented as mean ± standard error (*n* = 3 per treatment). Data were analyzed using one-way ANOVA with Tukey’s post hoc comparisons.

**Table 5:** Effect of sodium selenite and hydroxy-selenomethionine supplementation on growth performance in Hanwoo calves.

Item	Control	SS ^1^	OH-SeMet ^2^	*p*-Value
Body weight (kg)				
Birth	25.80 ± 3.36 ^b^	27.91 ± 4.50 ^ab^	31.20 ± 2.70 ^a^	0.009
3 months of age	97.46 ± 11.68	105.03 ± 12.42	109.82 ± 14.34	0.106
ADG (kg/d)	0.80 ± 0.11	0.86 ± 0.11	0.88 ± 0.15	0.291
Intake (DM kg/d)				
Concentrate	0.62	0.64	0.72	-
Timothy	0.19	0.23	0.22	-
Dry matter intake	0.81	0.87	0.94	-
Feed conversion rate	1.04 ± 0.15	1.03 ± 0.12	1.09 ± 0.16	0.545

^a.b^ Means with different superscripts in the same row are significantly different (*p* < 0.05). ^1^ SS: sodium selenite; ^2^ OH-SeMet: hydroxy-selenomethionine. Data are presented as mean ± standard error (*n* = 10 per treatment). Data were analyzed using one-way ANOVA with Tukey’s post hoc comparisons.

**Table 6:** Effect of sodium selenite and hydroxy-selenomethionine supplementation on plasma selenium, GPx and TIG concentrations in Hanwoo calves at 3 months of age.

Item	Control	SS ^1^	OH-SeMet ^2^	*p*-Value
Selenium (pg/mL)	85.62 ± 37.00	171.96 ± 117.13	205.41 ± 139.56	0.119
GPx ^3^ (ng/mL)	1.60 ± 0.43	1.95 ± 0.98	1.94 ± 0.84	0.600
TIG ^4^ (ng/mL)	10.63 ± 2.46	11.15 ± 2.22	13.02 ± 3.64	0.209
Leptin (ng/mL)	7.64 ± 0.24	7.72 ± 1.07	6.85 ± 0.98	0.100
Ghrelin (pg/mL)	19.57 ± 0.78 ^b^	19.13 ± 0.90 ^b^	23.53 ± 3.58 ^a^	<0.001
Growth hormone (pg/mL)	7.91 ± 2.54	7.50 ± 1.80	7.76 ± 2.19	0.925

^a,b^ Means with difference superscripts in the same row are significantly different (*p* < 0.05). ^1^ SS, sodium selenite; ^2^ OH-SeMet, hydroxy-selenomethionine; ^3^ GPx, glutathione peroxidase; ^4^ TIG, total immunoglobulin. Values are presented as means ± standard error (*n* = 10 per treatment). Data were analyzed using one-way ANOVA, and post hoc comparisons were conducted using Tukey’s test.

**Table 7:** Effect of sodium selenite and hydroxy-selenomethionine supplementation on plasma metabolite in Hanwoo calves at 3 months of age.

Items	Control	SS ^1^	OH-SeMet ^2^	*p*-Value
Glucose (mg/dL)	53.40 ± 8.87	55.00 ± 10.35	54.50 ± 7.20	0.919
Total protein (g/L)	44.10 ± 2.69	45.20 ± 3.05	44.50 ± 2.46	0.666
BUN ^3^ (mg/dL)	8.86 ± 2.24	9.23 ± 2.06	10.56 ± 2.37	0.219
Albumin (g/L)	22.90 ± 1.20	23.00 ± 1.15	23.10 ± 1.20	0.931
NEFA ^4^ (μEq/L)	338.50 ± 156.85	249.10 ± 86.99	210.70 ± 91.50	0.057
Triglyceride (mg/dL)	14.30 ± 6.17	12.80 ± 4.42	16.00 ± 4.64	0.391
Cholesterol (mg/dL)	84.60 ± 10.49 ^a^	65.60 ± 12.11 ^b^	62.50 ± 21.21 ^b^	0.007
Creatinine (mg/dL)	0.73 ± 0.07	0.74 ± 0.10	0.76 ± 0.08	0.734
AST ^5^ (U/L)	67.80 ± 28.49	50.00 ± 9.21	53.56 ± 8.46	0.103
ALT ^6^ (U/L)	8.50 ± 2.17	9.60 ± 1.90	9.10 ± 1.60	0.444
GGT ^7^ (U/L)	13.20 ± 6.86	12.67 ± 3.12	22.90 ± 14.10	0.061
Ca ^8^ (mg/dL)	6.22 ± 0.29	6.17 ± 0.14	6.24 ± 0.31	0.828
IP ^9^ (mg/dL)	5.20 ± 0.46	5.09 ± 0.29	5.19 ± 0.38	0.778
Mg ^10^ (mg/dL)	1.31 ± 0.11	1.26 ± 0.10	1.31 ± 0.07	0.407

^a.b^ Means with different superscripts in the same row are significantly different (*p* < 0.05). ^1^ SS, sodium selenite; ^2^ OH-SeMet, hydroxy-selenomethionine; ^3^ BUN, blood urea nitrogen; ^4^ NEFA, non-esterified fatty acid; ^5^ AST, aspartate-aminotransferase; ^6^ ALT, alanine aminotransferase; ^7^ GGT, gamma-glutamyl transferase; ^8^ Ca, calcium; ^9^ IP, inorganic phosphate; ^10^ Mg, magnesium. Data are presented as mean ± standard error (*n* = 10 per treatment). Data were analyzed using one-way ANOVA with Tukey’s post hoc comparisons.

## Data Availability

The original contributions presented in the study are included in the article and Supplementary Material.

## Supplementary Materials

The following supporting information can be downloaded at: https://www.mdpi.com/article/10.3390/ani16142258/s1, Figure S1: Effect of sodium selenite and hydroxy-selenomethionine supplementation on plasma selenium and glutathione peroxidase concentrations in Hanwoo cows at 3 months before and after calving. ^1^NEFA: Non-esterified fatty acid; ^2^BUN: Blood urea nitrogen; ^3^AST: Aspartate-amino-transferase; ^4^ALT: Alanine aminotransferase; ^5^GGT: Gamma-glutamyl-transferase; ^6^Ca: calcium; ^7^IP: Inorganic phosphate; ^8^Mg: magnesium; ^9^SS: sodium selenite; ^10^OH-SeMet: hydroxy-selenomethionine. Data are presented as mean ± standard error (*n* = 10 per treatment). Data were analyzed using two-way repeated-measures ANOVA with Tukey’s post-hoc test.

## Abbreviations

The following abbreviations are used in this manuscript:

ADG	Average daily gain
ALT	Alanine aminotransferase
AST	Aspartate aminotransferase
BUN	Blood urea nitrogen
FCR	Feed conversion ratio
GGT	γ-glutamyl transferase
GPx	Glutathione peroxidase
IP	Inorganic phosphorus
NEFA	Non-esterified fatty acid
OH-SeMet	Hydroxy-selenomethionine
SS	Sodium selenite
TIG	Total immunoglobulin
