# Supplementary material for: Effect of Sodium Selenite and Hydroxy-Selenomethionine Supplementation in Hanwoo Cows on Reproductive Performance and Growth Performance of Their Offspring
Source: Animals (Basel). 2026 Jul 21;16(14):2258. doi: 10.3390/ani16142258 (PMC13405943; doi:10.3390/ani16142258)
Supplement: Supplementary file 1 [file animals-16-02258-s001.zip › animals-4397002-supplementary.pdf]

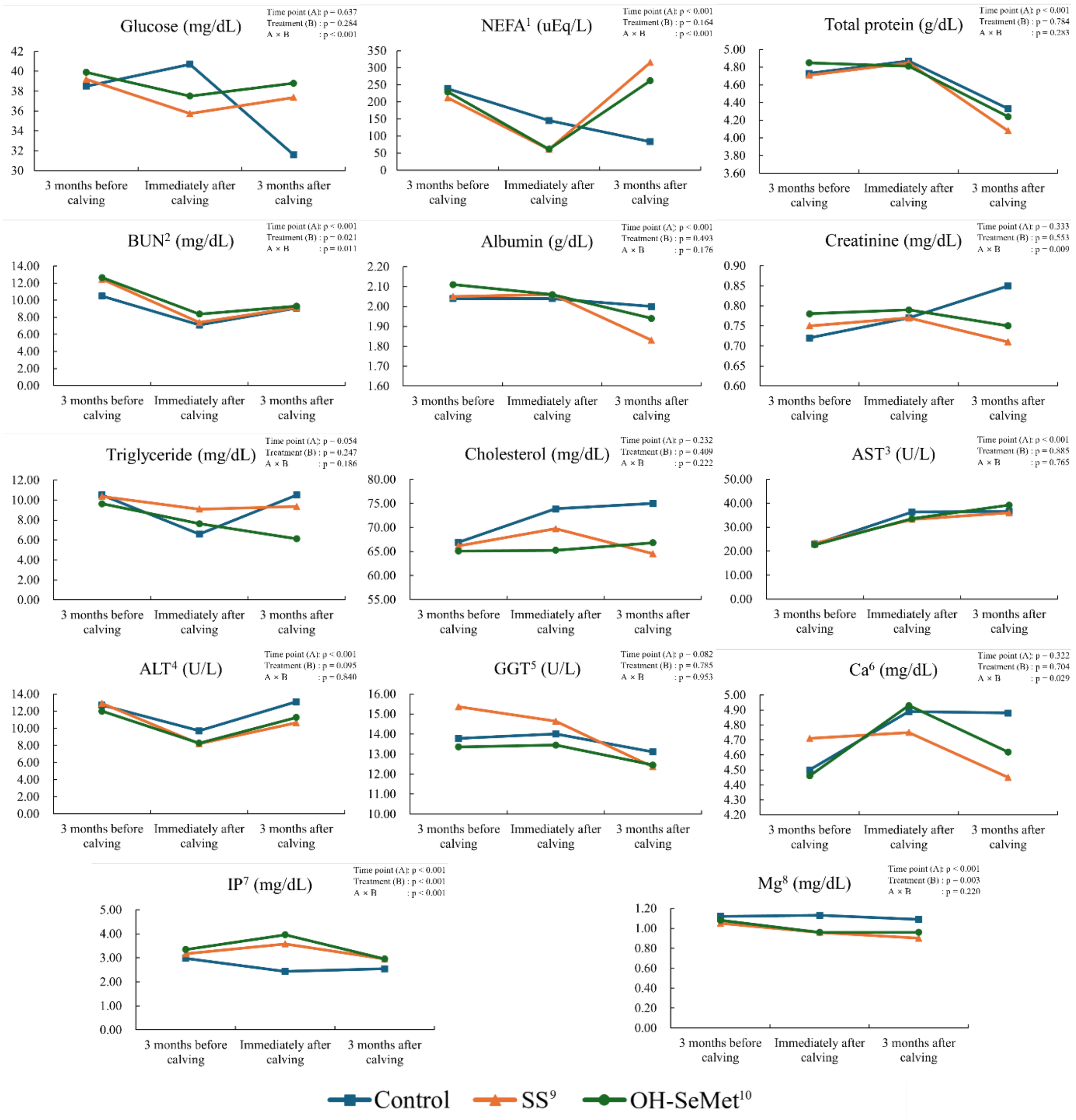

Figure S1. Effect of sodium selenite and hydroxy-selenomethionine supplementation on plasma selenium and glutathione peroxidase concentrations in Hanwoo cows at 3 months before and after calving. [1]NEFA: Non-esterified fatty acid; [2]BUN: Blood urea nitrogen; [3]AST: Aspartate-amino-transferase; [4]ALT: Alanine aminotransferase; [5]GGT: Gamma-glutamyl-transferase; [6]Ca: calcium; [7]IP: Inorganic phosphate; [8]Mg: magnesium; [9]SS: sodium selenite; [10]OH-SeMet: hydroxy-selenomethionine. * Data are presented as mean ± standard error (n = 10 per treatment). ** Data were analyzed using two-way repeated-measures ANOVA with Tukey's post-hoc test.
